# Effect of α-Hemolysin Producing *E. coli* in Two Different Mouse Strains in a DSS Model of Inflammatory Bowel Disease

**DOI:** 10.3390/microorganisms8121971

**Published:** 2020-12-11

**Authors:** Hengameh Chloé Mirsepasi-Lauridsen, Carsten Struve, Andreas Munk Petersen, Karen Angeliki Krogfelt

**Affiliations:** 1 Department of Bacteria, Parasites and Fungi, Statens Serum Institut, 2300 Copenhagen, Denmark; struvec@yahoo.dk; 2 Department of Biology, University of Copenhagen, 2200 Copenhagen, Denmark; 3 Division of Gastroenterology, BC Children’s Hospital, University of British Columbia, Vancouver, BC V6H 3N1, Canada; 4 Institute of Molecular and Medical Biology, Roskilde University, 4000 Roskilde, Denmark; karenak@ruc.dk; 5 Department of Gastroenterology, Copenhagen University Hospital Hvidovre, 2650 Hvidovre, Denmark; andreas.munk.petersen@regionh.dk; 6 Department of Clinical Microbiology, Copenhagen University Hospital Hvidovre, 2650 Hvidovre, Denmark; 7 Department of Virus & Microbiological Special Diagnostics, Statens Serum Institute, 2300 Copenhagen, Denmark

**Keywords:** inflammatory-bowel-disease, Sigirr −/− mice, C57BL/6-mice, *Escherichia coli*, alpha hemolysin

## Abstract

Background: Phylogroup B2 *Escherichia coli* have been associated with ulcerative colitis (UC). In this study, we aimed to compare colonization with the UC-associated *E. coli* p19A in different mice strains, to investigate the role of alpha hemolysin in a UC mouse model. Methods: In this study, Sigirr −/− and C57BL/6 mice were chosen, and UC was induced by adding dextran sulfate sodium (DSS) to the drinking water. The mice were pre-treated with ciprofloxacin. p19A expressing luminescence and GFP, alpha-hemolysin knock out p19A-ΔhlyI II, and non-pathogenic lab *E. coli* DH10B were cultured in LB broth, and orally gavaged into the mice. Colonization with p19A WT was visualized using an in vivo imaging system. Results: p19A WT colonized the colon, ileum, Peyer’s patches, liver, and spleen of infected C57BL/6 and Sigirr −/− mice. A total of 99% of the p19A WT infected C57BL/6 mice and 29% of the p19A WT infected Sigirr −/− mice survived to the 4th post infection day. Conclusion: UC-associated *E. coli* p19A WT colonized the intestines of DSS-treated mice and caused extra-intestinal infection. Hemolysin is an important factor in this pathogenesis, since isogenic hemolysin mutants did not cause the same inflammation.

## 1. Introduction

Inflammatory bowel diseases (IBD) can be divided into ulcerative colitis (UC) and Crohn’s disease (CD). CD can affect any part of the gastrointestinal tract, while UC is restricted to the colon and, during flares of the disease, is characterized by bloody diarrhea [1]. The etiology of IBD is unknown, but immunological investigations of animal models have shown that intestinal inflammation is derived from changes in the intestinal microbiota [2], triggering an abnormal immune response [3]. These abnormal immune responses to intestinal microbiota changes in IBD cause activation of innate immune receptors such as Toll-like receptors (TLRs), which leads to upregulation of antimicrobial factors, and secretion of cytokines and chemokines [4]. Sigirr is an IgG IL-1-related receptor suspected to play a role in the pathogenesis of IBD [5]. Sigirr is a negative regulator of TLRs expressed by intestinal epithelial cells, and regulates inflammation and mucosal homeostasis; in a mouse model, deletion of the Sigirr gene has been used to enhance chemical colitis [6,7].

There are five different categories of IBD animal models: (1) antigen-induced colitis and colitis induced by microbiota, (2) adaptive infection models, (3) chemically induced colitis, (4) genetically modified colitis models, (5) and spontaneous colitis models [8]. However, studies have indicated that germfree animals generally do not develop intestinal inflammation, and that spontaneous gut inflammation requires a certain genetic background [9]. This suggests that the nexus of IBD pathogenesis lies in the interaction between predisposing host genetic factors and the host immune response to intestinal bacteria.

Roland Bücker et al., 2019 [10], showed that colitis-susceptible IL 10 −/− mice colonized with an HlyA-expressing *Escherichia coli* (*E. coli*) had elevated inflammation scores and an increased epithelial permeability compared with mice colonized with an HlyA-deficient mutant.

Placebo-controlled studies have shown that antibiotic treatment can induce remission in UC patients [11,12]. *E. coli* has been suspected since the 1970s as the reason for relapses in UC [13]. Many studies have demonstrated an increased prevalence of *E. coli* with virulence properties in UC patients, especially within UC disease relapses [14,15,16]. Bacteriological analysis of biopsies and fecal samples from UC patients tends to show an increased prevalence of *E. coli* species belonging to the B2 phylogenetic groups, harboring extra-intestinal pathogenic *E. coli* (ExPEC) genes [17,18]. Diffusely adherent *E. coli* has been linked to UC [19], while adherent invasive *E. coli* has been linked to CD [20].

Previous studies have shown that the UC-associated *E. coli* p19A WT from the B2 phylogenetic group, harboring 2 alpha-hemolysins, induces cell death in dendritic cells (DC) and stimulates TNF-α, IL-6, and IL-23 [21]. UC-associated *E. coli* p19A WT has been shown to dissolve occludin, and thereby disrupt tight junctions in Caco-2 cells in vitro and increase barrier permeability [22]. In this study, the aim was to compare colonization with p19A WT in DSS-treated Sigirr −/− and C57BL/6 mice, and to evaluate the pathogenic features of UC-associated *E. coli* p19A WT in the intestines of infected mice in comparison to mice colonized with the non-pathogenic *E. coli* DH10B. The second aim of this study was to investigate the role of the *E. coli* alpha hemolysin gene in an IBD mouse model, by colonizing the mice with double alpha hemolysin gene knock out p19A (p19A ΔhlyI, II).

## 2. Results

### 2.1. Course of Infection of Ecoli in a DSS Mouse Model

**Figure 1:**
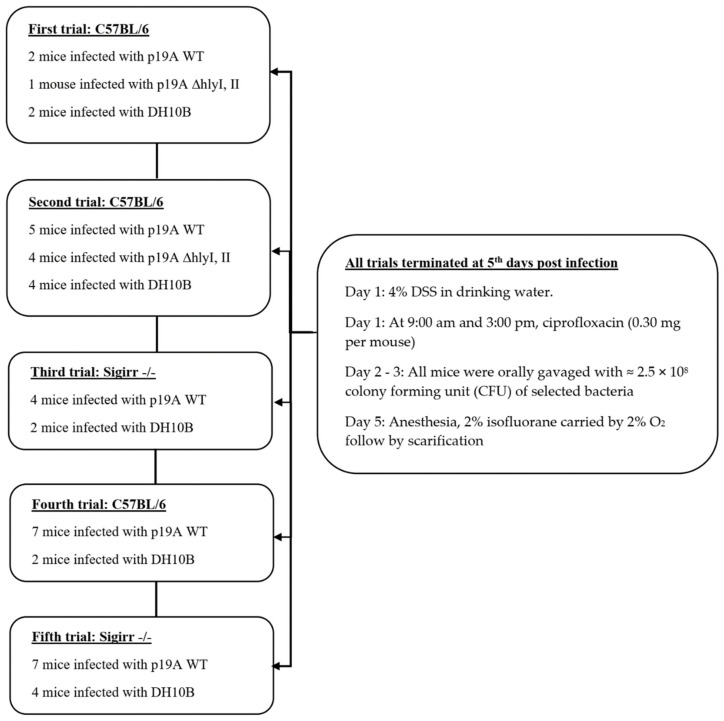
The picture shows a flow diagram of the infection course in Sigirr −/− and C57BL6 mice. The first trial with C57BL/6 mice was performed to optimize the dosage of antibiotic, dextran sulfate sodium (DSS), and amount of bacteria used to colonize the mice in the experimental group. The second and fourth trials were the same experiment performed twice, to control the outcome in the C57BL/6 mice model. Trials 3 and 5 were the same experiment performed twice, to control the outcome in the Sigirr −/− mice model.

The first trial was performed to find optimal dosage of ciprofloxacin and DSS and amount of inoculation of the bacteria in experimental mice. In this study, we performed five experiments in total (Figure 1). The second and fourth trial was performed to discover the pathogenesis of p19A WT and p19A ΔhlyI, II colonization in the dextran sulfate sodium (DSS) treated C57BL/6 mice. The third and the fifth trial was performed to discover the pathogenesis of p19A WT colonization in DSS treated Sigirr −/− mice (Figure 1). In all the trials, after first post-infection day, mice infected with p19A WT showed signs of being hunched, sometimes with a “round” appearance, and reluctance to move, with ruffled fur and squinting eyes.

**Figure 2:**
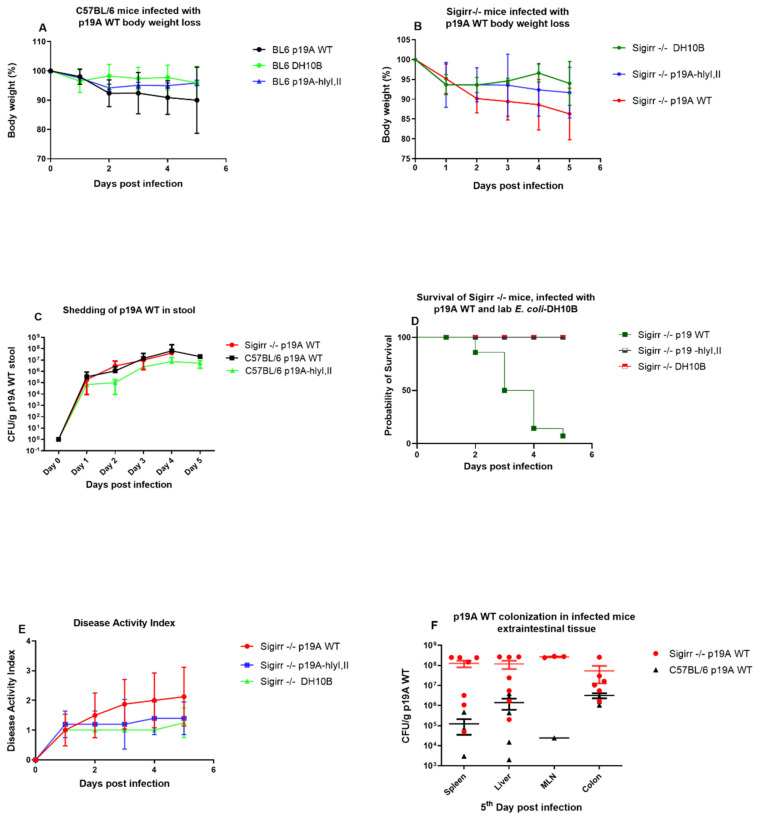
p19A WT infected mice: colonization and the course of infection. (**A**) p19A WT infected C57BL/6 mice show ≤ 10% body weight loss, while the p19A ΔhlyI, II and DH10B infected group had ≤5% body weight loss at day 5 post-infection, *p* < 0.05. (**B**) p19A WT infected Sigirr −/− mice showed ≤13.7% (mean value) body weight loss, while DH10B and p19A ΔhlyI, II infected mice had ≤6% and ≤8% (mean value) body weight loss, respectively at day 5 post-infection, *p* < 0.0037. (**C**) There were no differences in shedding of p19A WT and p19A ΔhlyI, II in the stool of infected C57BL/6 and Sigirr −/− mice at the 5th day post-infection (Kruskal–Wallis non-parametric test). (**D**) Survival analysis of p19A WT infected Sigirr −/− mice showed that 29% of the infected mice survived at 4th day post-infection, while 100% of the Sigirr −/− mice infected with p19A ΔhlyI, II and DH10B survived until the 5th day post infection. (**E**) Disease activity index (DAI) for Sigirr −/− mice infected with p19A WT (DAI, mean ≈ 2.3), p19A ΔhlyI, II and DH10B (DAI, mean ≈ 1.2), and C57BL/6 mice infected with p19A WT (DAI, mean ≈ 2.5), at the 5th day post-infection. (**F**) Liver, spleen, metastatic lymph nodes (MLN) and colon of p19A WT infected C57BL/6 1.4 × 10^6^ to 1.1 × 10^5^) and Sigirr −/− mice, 1.1 × 10^8^, were colonized with p19A WT at day 5 post-infection. The CFU in the liver and spleen, mean value 1.1 × 10^5^ to 6.3 × 10^5^ and 4.3 × 10^3^ to 4.3 × 10^5^, respectively.

C57BL/6 mice infected with p19A WT had a ≤10% (mean value) body weight loss (Figure 2). C57BL/6 mice infected with DH10B and p19A ΔhlyI, II had ≤ 4% and ≤6% (mean value) body weight loss, respectively. The experiment was ended at the 5th post-infection day. Sigirr −/− mice infected with p19A WT had ≤13.7% (mean value) body weight loss and had a mean disease activity index (DAI) of 2.3 at the 5th post-infection day (Figure 2). DH10B and p19A ΔhlyI, II infected Sigirr −/− mice had ≤ 6% and ≤8% (mean value) body weight loss, respectively, *p* < 0.0037 **. From 1 to 5 days post-infection, p19A WT was found in stools of infected C57BL/6 and Sigirr −/− mice at ≈10^7^ colony forming units (CFUs) (Figure 2). Some Sigirr −/− mice became very sick after p19A WT inoculation and died after ≈3 days; 86% of the 10 Sigirr −/− mice infected with p19A WT survived at day 2, 64% survived at day 3, and 29% survived at day 4 post-infection (Figure 2). However, 99% of the p19A WT infected C57BL/6 mice survived to the 5th post-infection day, **** *p* < 0.0001. The CFU of the systemic organs showed that p19A WT colonized the livers and spleens of infected Sigirr −/− and C57BL/6 mice (Figure 2). However, the colonization of p19A WT in the livers and spleens of Sigirr −/− mice was much greater (liver and spleen-median ≈ 1.1 × 10^8^) than in C57BL/6 mice (liver ≈ 1.4 × 10^6^, spleen-median ≈ 1.1 × 10^5^) (Figure 2). p19A WT also colonized the colon and metastatic lymph nodes (MLN) of Sigirr −/− mice and C57BL/6 mice (Figure 2). However, the Sigirr −/− mice infected with DH10B and p19A ΔhlyI, II survived (100%), and had a mean DAI of 1.2 at the 5th post-infection day (Figure 2).

The CFU of C57BL/6 mice infected with p19A ΔhlyI, II showed ≈10^6^ shedding in the stool at the 4th post-infection day (Figure 2). C57BL/6 mice infected with non-pathogenic *E. coli*, DH10B, and p19A ΔhlyI, II had a ≤5% body weight loss (Figure 2).

### 2.2. Macroscopic and In Vivo Imaging System

**Figure 3:**
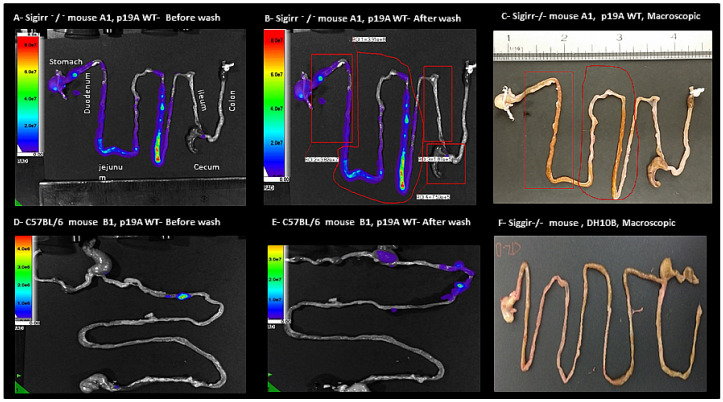
In Vivo Imaging System (IVIS) evaluation of experimental mice. (**A**) p19AWT infected Sigirr −/− mouse on the 3rd day post-infection, expressing 50× greater luminescent signals from the gastrointestinal-tract before washing (**B**) and after washing in phosphate-buffered saline (PBS). (**C**) Macroscopic picture of the gastrointestinal tract of the same mouse from Figure 3A, showing the area of inflammation as thickened, swollen tissues marked with red. (**D**) p19A WT infected C57BL/6 mouse on the 4th day post-infection, expressing luminescent signals from the colon, cecum and ileum before washing (**E**) and after washing in PBS. (**F**) Macroscopic picture of the gastrointestinal tract of a DH10B infected mouse, showing no signs of inflammation or thickened or swollen tissues.

After anesthesia of the infected Sigirr −/− and C57BL/6 mice, In Vivo Imaging System (IVIS) imaging was performed. IVIS images of infected mice showed p19A WT in the intestine (Figure 3, while uninfected mice, as expected, showed no signals. Infected Sigirr −/− mice showed up to 50× greater luminescent signals in the ileum, jejunum and duodenum, before and after washing in phosphate-buffered saline (PBS) (Figure 3). in comparison top19A WT infected C57BL/6 mice, in which signals only occurred in the colon and ileum. Macroscopic pictures of the intestine of p19A WT infected Sigirr −/− mice showed a thickened and yellowish/swollen ileum and jejunum (Figure 3). p19A ΔhlyI, II, DH10B, and non-infected Sigirr −/− and C57BL/6 mice showed no similar macroscopic changes (Figure 3).

**Figure 4:**
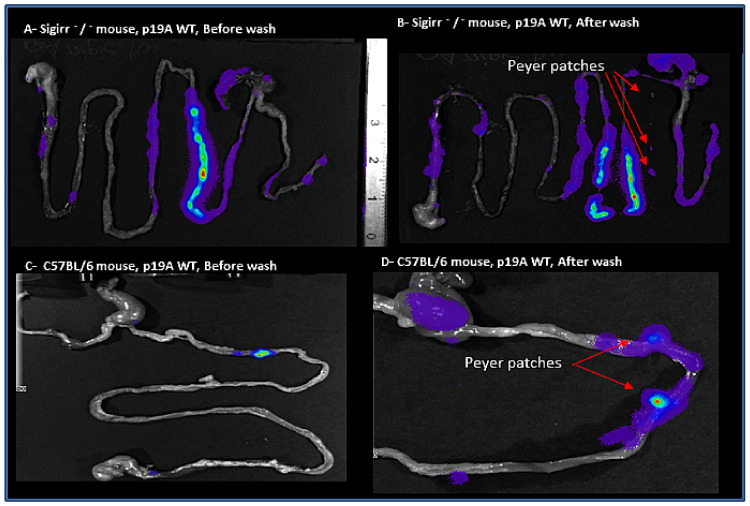
Macroscopic evaluation of the tissues. (**A**) p19A WT infected Sigirr −/− mouse on the 4th day post-infection, expressing luminescent signals from the gastrointestinal tract. (**B**) p19A WT infected Sigirr −/− mouse on the 4th day post-infection, after washing in PBS, expressing luminescent signals from Peyer’s patches, shown with red arrows. (**C**) p19A WT infected C57BL/6 mouse on the 3rd day post-infection, expressing luminescent signals from the ileum. (**D**) p19A WT infected C57BL/6 mouse on the 3rd day post-infection, after washing in PBS, expressing luminescent signals from Peyer’s patches, shown with red arrows.

IVS imaging showed increased colonization with p19A WT in Peyer’s patches of Sigirr −/− and C57BL/6 mice before and after washing, respectively (Figure 4).

### 2.3. Histology

**Figure 5:**
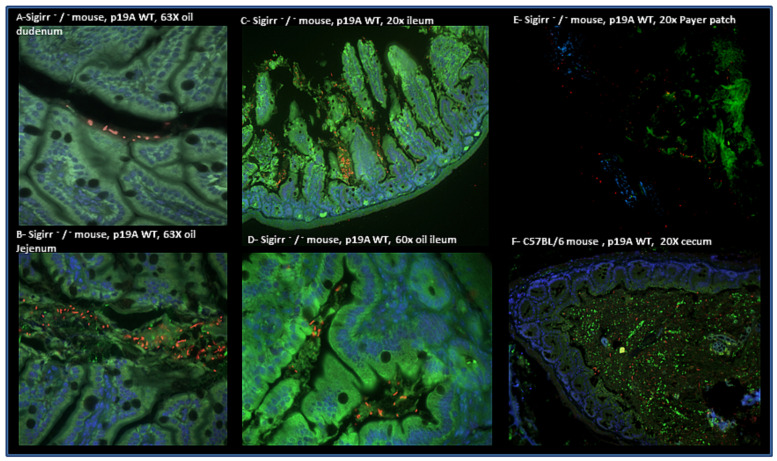
Immunofluorescence staining of the experimental mouse intestinal tissues. (**A**) p19A WT (in red) is shown in the duodenum of a p19A WT infected Sigirr −/− mouse. (**B**) p19A WT (in red) is shown in the jejunum of an infected Sigirr −/− mouse. (**C**) p19A WT (in red) is shown in the ileum of an infected Sigirr −/− mouse. (**D**) p19A WT (in red) is attached to epithelial cells along the ileal crypt/villus in an infected Sigirr −/− mouse. (**E**) Immunofluorescence staining of the Peyer patches of a p19A WT infected Sigirr −/− mouse, showing p19A WT in red attached to the Payer’s patches. (**F**) p19A WT in red, shown in the cecum of an infected C57BL/6 mouse.

Immunofluorescence staining showed that p19A WT, in red, colonized the mucus layer of the jejunum, duodenum and ileum, and attached to epithelial cells along the ileal crypt-villus (Figure 5) and in the Peyer’s patches of infected Sigirr −/− mice (Figure 5). However, p19A WT (in red) was mostly attached to the colon and cecum of infected C57BL/6 mice (Figure 5).

**Figure 6:**
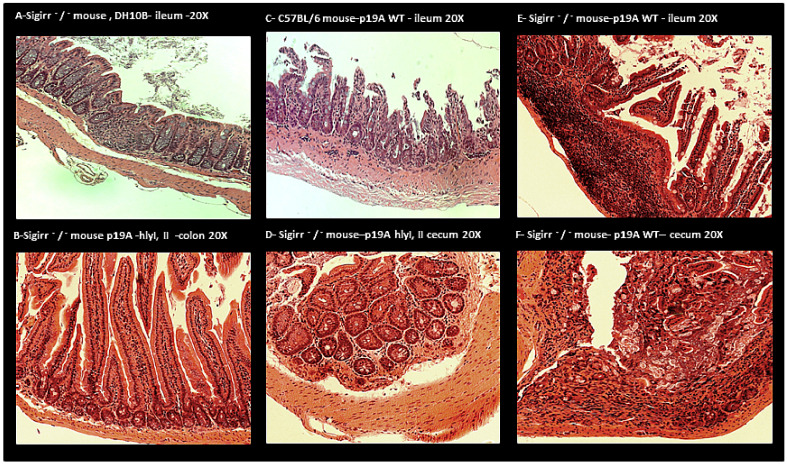
The histology/hematoxylin and eosin (HE) staining of the intestinal tissue of colonized mice. (**A**) DH10B infected Sigirr −/−mouse, showing no signs of tissue damage in the ileum. (**B**) p19A ΔhlyI, II infected Sigirr −/− mouse showing no signs of tissue damage in the colon. (**C**) p19A WT infected C57BL/6 mouse showing inflammation/increased leucocytes and thickness of smooth muscle and ulcer formation in the ileum. (**D**) p19A ΔhlyI, II infected Sigirr −/−mouse showing no signs of tissue damage or inflammation in the cecum. (**E**) p19A WT infected Sigirr −/− mouse showing inflammation/increased leukocytes in the Peyer patches and tissue damage. (**F**) p19A WT infected Sigirr −/− mouse showing inflammation/increased leucocytes and initiation to ulcerative formation in the cecum.

Hematoxylin and eosin (HE) staining of Non-pathogenic *E. coli* DH10B (Figure 6) and p19A ΔhlyI, II infected C57BL/6 (Figure 6) and Sigirr −/− mice showed no sign of inflammation in their intestine.HE staining of p19A WT infected Sigirr −/− and C57BL/6 mice showed increased inflammation/increased leucocytes, thickness of smooth muscles and initiation to ulcerative formation in the ileum and cecum of the infected mice, to a higher degree in Sigirr −/− mice than in C57BL/6 mice (Figure 6).

## 3. Discussion

*E. coli* harboring extra pathogenic *E. coli* (ExPEC) genes, such as alpha hemolysin, has been associated with IBD patients with active disease [8,21]. *E. coli* strain p19A, harboring ExPEC genes such as alpha hemolysin, was isolated from a fecal sample of an IBD patient with active disease. In the present study, the goal was to investigate if *E. coli* p19A would be able to colonize the gastro-intestinal tract and extra intestinal tissue of infected mice in a UC mouse model.

As described previously, IBD animal models are divided into five categories, however chemically induced colitis using DSS is widely used to study UC, wherein inflammation occurs in the colon of the mice [23]. Some studies suggest using ghrelin in mice-treated with DSS, since it has protective and therapeutic effects in the gut of DSS treated mice and limits tissue damage [24]. However, it was not necessary in our DSS-mouse model, since the control group, which was treated with commensal *E. coli* DH10B, did not show any signs of sickness/stress and the histology pictures of their tissues showed no signs of inflammation. This might be due to experiment duration, which was only 5 days.

Gene knock-out models are also widely used, such as the IL-10 −/− model, where inflammation occurs in the colon of the mice. IL-10 is a pivotal cytokine that maintains a check on pro-inflammatory responses to normal antigens and beneficial bacteria. Deficiencies in IL-10 are suspected to play a role in IBD pathogenesis.

In this study, we used C57BL/6 and Sigirr −/− mice, and UC was induced by adding dextran sulfate sodium (DSS) to their drinking water. Sigirr −/− mice, deficient in SIGIRR, belonging to the interleukin-1 receptor family [5], were chosen since SIGIRR is suspected to play a role in IBD [7,25]. To achieve optimal colonization with p19A WT in the UC mouse model, with reduced competition from already existing mouse-intestinal-bacteria, pre-treatment with antibiotics was necessary.

Sigirr −/− and C57BL/6 mice infected with p19A WT showed signs of sickness, to a higher degree in the Sigirr −/− mice than in the C57BL/6 mice. This was confirmed by IVIS, wherein p19A WT infected Sigirr −/−mice showed up to 50 × greater luminescent signals from intestinal tissues in comparison to C57BL/6 mice. The macroscopic pictures of the intestines of Sigirr −/− mice infected with p19A WT showed heavily thickened and inflamed/swollen ileums and jejunums. These results indicate that p19A WT colonizes the intestines of both UC mouse phenotypes, and causes disease/infection and ulceration of the intestinal tissue, as is characteristic for IBD. However, it is still uncertain if disease relapses in UC are caused by infection with *E. coli* species harboring ExPEC genes.

It is well known that inflammation causes increased release of nonfermentable nitrate, which serves as a substrate for *E. coli* nitrate respiration, enabling overgrowth of “commensal” *E. coli* species in the lumen of an inflamed gut [26]. IBD animal models have clarified that gut bacteria play an essential role in development of intestinal inflammation, since germfree animals do not generally develop intestinal inflammation [9]. Bacterial colonization in Sigirr −/− mice indicates small intestinal bacterial overgrowth (SIBO), which is often linked to Crohn’s disease (CD), where increased *E. coli* colonization was shown in the jejunum and decreased *E. coli* colonization was observed in the colon. However, these changes were followed by changes in gastrointestinal PH, which create an optimal environment for species such as *E. coli* to colonize and overgrow [27]. Another way of looking at this phenomenon is by considering oxidative stress, which is linked to IBD. Reactive oxygen species (ROS) such as H_2_O_2_ are distributed in the human liver and kidney. An alteration of body antioxidant defense mechanisms has been linked to some pathogenic bacteria, such as *Escherichia coli*, *Shigella*, *Salmonella*, *Campylobacter jejuni* and *Helicobacter pylori*. However, reduced occurrence of antioxidant defense mechanisms, such as catalase, is linked to CD and colon cancer [28,29]. More investigation is needed to clarify the reduced occurrence of antioxidant defense mechanisms in IBD, and if there is a link between increased ROS and prevalence of intestinal bacterial dysbiosis in IBD patients.

CD is a chronic, segmental, localized granulomatous disease that can affect all parts of the gastrointestinal tract [1], and is linked to an immunologic and genetic defect [30,31]. Our results indicate that DSS treated Sigirr −/− mice, which are deficient in SIGIRR/in interleukin-1 receptor, serve as a good model for CD, where inflammation/infection occurs in the small intestine and Peyer’s patches, in comparison to the other IBD in vivo model, where inflammation occurs only in the colon. However, C57BL/6 mice would seem a better model for UC, where inflammation is mostly restricted to the colon and cecum. Immunofluorescence staining of p19A WT infected Sigirr −/− mice showed increased colonization of Peyer’s patches, a phenomenon linked to CD. Benoit Chassaing et al., 2011 [32], showed that adherent-invasive *E. coli* (AIEC) via long polar fimbriae (lpf) expressing type 1 pili colonize the Peyer’s patches of infected mice. Previous studies have shown that *E. coli* p19A WT does not harbor lpf/lpf2 genes [33]. Julien Matricon reported increased inflammation/infection of Peyer’s patches in CD patients, caused by bacterial translocation of *E. coli* species [34]. This result suggests that lpf is not the only pathway used by *E. coli* to adhere to and colonize Peyer’s patches in mice.

Sigirr −/− mice infected with p19A WT only had a survival of 64% at 3 days post-infection, which indicates blood poisoning followed by septic shock of the infected mice, *p* < 0.0001 ***. The CFU of the extra-intestinal tissues of p19A WT infected Sigirr −/− and C57BL/6 mice showed p19A WT colonized the MLN, liver and spleen of the infected mice. These results indicate that p19A WT behaves like ExPEC [35] and infects intestinal and extra-intestinal tissues. P19A WT harbors two alpha-hemolysin genes, which disrupt tight junctions in the intestinal epithelial cells, leading to increased epithelial permeability and bacterial translocation to extra-intestinal tissues. This was confirmed by mice infected with p19A ΔhlyI, II, where both alpha-hemolysin genes were knocked out. Histology of intestinal tissues of p19A ΔhlyI, II infected mice showed no signs of inflammation.

This study indicates that the Sigirr −/− mouse is a good model to study CD, as the inflammation occurs in the entire gastrointestinal tract and Peyer’s patches, as in humans. We also show that IBD associated *E. coli*, p19A WT harboring alpha hemolysin genes causes intestinal and extraintestinal inflammation and promotes IBD characteristic pathogenesis in an infected IBD-mouse-model. This finding suggests that IBD associated *E. coli* might play a role in IBD disease relapses.

## 4. Conclusions

UC-associated *E. coli* p19A WT colonizes the intestines of DSS-treated mice and causes extra-intestinal infection especially in the Sigirr −/− mouse. Hemolysin is an important factor in the pathogenesis, since isogenic hemolysin mutants did not cause the same degree of inflammation. The Sigirr −/− mouse is a better model than the C57BL/6 mouse to study CD, where infection/inflammation occurs in the ileum/small intestine and Peyer’s patches. However, the DSS-treated C57BL/6 mouse is a good model to study UC, with infection/inflammation in the cecum and colon.

## 5. Materials and Methods

### 5.1. Clinical Isolate and Generated Mutants Used

In this study we use a clinical fecal *E. coli* from the B2 phylogenetic group, isolated from a patient with UC [18]. The parental p19A WT and its derivative strain, p19 ΔhlyI, II, lacking both alpha hemolysin genes, have both been described previously [16].

To facilitate the recovery of p19A from tissues or feces, we generated a p19A derivative strain expressing chloramphenicol resistance on the chromosome of p19A WT, as described previously. p19A WT, *E. coli* MFDλ*pir* containing pMAC5 (a chloramphenicol-marked Tn7 delivery vector) [36,37], and MFDλ*pir* containing a helper plasmid pTNS2 [38], were conjugated on LB agar containing diaminopimelic acid (DAP). After 24 h, the conjugation mixture was eluted and plated onto LB agar containing chloramphenicol but lacking DAP. The p19A WT containing the proper Tn7 transposition was checked as before [38], and a p19A WT-lux strain was generated expressing the *Photorhabdus luminescens* lux operon [39]. The lux operon was expressed under the control of the promoter PLtetO [40].

To create a p19A WT expressing green fluorescent protein (GFP), an Enteropathogenic *E. coli* strain harboring GFP expressing plasmid (PFVP25, 4764 bp, resistance to streptomycin) was used.

All bacterial strains were grown from single colonies on LB plates, and cultured in LB broth with chloramphenicol (30 μg/mL) or streptomycin (50 μg/mL) at 37 °C overnight with shaking.

### 5.2. Infection Course in DSS Mouse Model

Six-to-ten-week-old male C57BL/6 (WT) and Sigirr −/− mice (Charles River Laboratories breeders: Wilmington, MA, USA) were bred in-house. The experiment was repeated with 2–7 mice in each group, which were separated in different cages. In total 44 mice were used for this experiment, of which 17 were Sigirr −/− mice and 27 were C57BL/6 mice. All mice were housed individually in a temperature-controlled (22 ± 2 °C) animal facility, with a 12 h light-dark cycle. The mice were maintained under specific pathogen-free conditions at the Child and Family Research Institute.

On the first day of the experiment, at 9:00 a.m. and 3:00 p.m., the mice were pre-treated with ciprofloxacin (0.30 mg per mouse) and 4% DSS was added to their drinking water at 9:00 a.m. On the second and third day of the experiment, at 9:00 a.m. and at 3:00 p.m. each mouse was orally gavaged with ≈2.5 × 10^8^ colony forming unit (CFU) p19A WT-lux, p19A WT-GFP, p19A ΔhlyI, II and non-pathogenic lab-*E. coli* DH10B (Figure 1). The mice were euthanized on the 5th post-infection day. Colonization was monitored using an in vivo imaging system (before and after washing the luminal contents). Tissues were collected in formalin and 4% paraformaldehyde for histology, and in phosphate-buffered saline (PBS) for CFU analysis.

In total, 5 different trials were performed. The first trial was performed with B57BL/6 mice to discover the optimal dosage of DSS, ciprofloxacin and the amount of bacteria used to colonize the mouse. The first trial helped us to discover that the experiment has to end at the 5th day post-infection, since the mouse become very sick. The second and fourth trials were performed to discover the effect/pathogenesis of p19A WT and p19A ΔhlyI, II colonization in the C57BL/6 mice. The third and the fifth trails were performed to discover the effect/pathogenesis of p19A WT colonization in Sigirr −/− mice (Figure 1).

Disease activity index (DAI) scores were recorded according to the following criteria: score 0—no weight loss and hard stool; score 1—less than 10% body weight loss and hard stool; score 2—10–15% body weight loss, loose stool and fecal occult blood; score 3—15–20% body weight loss, loose stool and fecal occult blood; score 4—>20% body weight loss, diarrhea and gross blood.

### 5.3. Ethics Statement

The mice were fed a standard sterile chow (Laboratory Rodent Diet 5001, Purina Mills, MO, USA) as well as tap water ad libitum throughout the experiment. All procedures involving care and handling of the mice were performed according to protocol number A15-0206, approved by the University of British Columbia Animal Care Committee (4 March 2014) and in direct accordance with the Canadian Council of Animal Care (CCAC) guidelines. The mice were monitored daily for mortality and morbidity throughout their infection, and euthanized if they showed signs of extreme distress or body weight loss (>20%). All surgeries were performed under anesthesia (2% isofluorane carried by 2% O_2_), and all efforts were made to minimize suffering.

### 5.4. Imaging

In Vivo Imaging System (IVIS) (Xenogen, Almeda, CA, USA) grayscale reference images taken under low illumination were collected and overlaid with images capturing the emission of photons from the lux-expressing bioluminescent IBD-associated *E. coli* p19A WT, using LIVING IMAGE software (Xenogen) and Igor (Wavemetrics, Seattle, WA, USA). The images were taken after the mice were euthanized and stomach-intestinal organs were removed, emptied and washed in PBS.

### 5.5. Tissue Collection

Tissue collection and CFU were performed as described by Khan et al., 2008 [41]. The mice were euthanized, dissected and the intestines were collected in 10% neutral buffered formalin (Fisher) for histology. The liver and spleen were collected and weighed immediately, homogenized in PBS pH 7.4, plated on chloramphenicol resistance LB agar plates and incubated overnight at 37 °C and 5% CO_2_ for CFU analysis.

### 5.6. Histological and Immunofluorescence Staining

Histological analysis was performed as described by Khan et al., 2006 [42]. Immunofluorescence staining of not-infected and infected mouse intestinal tissues was performed in 10% formalin. Peyer’s patches were fixed in 4% PFA, washed in PBS, and embedded in an optimal cutting template compound, before being frozen with isopentane and liquid N_2_. Serial sections of six μm in thickness were cut for immunofluorescence staining.

Formalin-fixed tissues were deparaffinized. Both formalin and PFA-fixed tissues were rehydrated, before antigen retrieval using preheated buffer (8.0 mm sodium citrate, 0.05% Tween 20, pH 6.0) and being steamed for 30 min. Sections were cooled down for 30 min, then washed in water for 3 min. Two percent goat serum was used to block cells for 1 h at room temperature. Thereafter, sections were incubated in primary antibody ((cytokeratin 19 green, goat antibody, cat PM 007-05008, Vancouver, Canada) (*E. coli* LPS O6 antibody red, 85002 (SS) Statens Serum Institut, Copenhagen, Denmark)) overnight at 4 °C in the dark. The next day, the sections were washed three times for five 5 min each time in PBS, incubated for 1 h at room temperature in a secondary antibody ((Donkey anti goat, Alexa fluor 488 Invitrogen, USA) (Donkey anti-rabbit IgG antibodies, Alexa fluor 568, Life technologies, CA, USA)), then washed two times for 5 min each time in PBS followed by 5 min in water. Then, the liquid mountant was applied directly to fluorescently labeled tissue samples on microscope slides (Prolong Gold antifade regent containing 42, 62-diamidino-2-phenylindole (DAPI) (Invitrogen)). Tissues were visualized at 350 and 594 nm using a Lecia DM 4000B microscope equipped with a Retiga 1300i Ast camera (Olmaging, Burnaby, BC, Canada), operating through Open Laboratory software 4.0.2. Hematoxylin and eosin (HE) staining was used for evaluating the tissues/histology.

### 5.7. Statistical Analysis

For this study, GraphPad Prism version 8.4.3. was used. CFU comparison analysis was performed using the Kruskal–Wallis non-parametric test with mean values and standard deviations for each time point/group. A two way ANOVA test was used to compare the body weight loss and CFU from extraintestinal tissue, with mean values and standard deviations for each time point/group. For survival analysis, the Gehan–Breslow–Wilcoxon test was used. *p* < 0.05 was considered significant.

## Abbreviations

IBD	Inflammatory bowel disease
GIT	Gastrointestinal tract
UC	Ulcerative colitis
CD	Crohn’s disease
ExPEC	Extra-intestinal pathogenic *E. coli*
AIEC	Adherent-invasive *E. coli*
DC	Dendritic cells
DSS	Dextran sodium sulfate
*E. coli*	*Escherichia coli*
